# Analysis of covariance and sample size calculation for comparing means in randomized controlled trials

**DOI:** 10.34172/jcvtr.2023.30497

**Published:** 2023-06-29

**Authors:** Yousef Alimohamadi, Mojtaba Sepandi

**Affiliations:** Health Research Center, Life Style Institute, Baqiyatallah University of Medical Sciences, Tehran, Iran

## Dear Editor,

We read the valuable manuscript with the title: Effects of sodium selenite and selenium-enriched yeast on cardiometabolic indices of patients with atherosclerosis: A double-blind randomized clinical trial study that published in J Cardiovasc Thorac Res.^1^ Which seems to have shortcomings in the following cases: Firstly, about determined sample size the authors mentioned “ The sample size of the study was defined based on the mean ( ± standard deviation [SD]) of GPX with a confidence interval of 95% and power of 80 %, and estimation 10% of dropouts.” The authors used the sample size formula for estimation a single mean which is not applicable for analytical studies such as randomized control trials. The appropriate formula of sample size for comparing two means is as follows:


$$N=\frac{2{\left(z_{1-\alpha /2}+z_{1-\beta }\right)}^{2}s_{p}^{2}}{\delta^{2}}$$

Where δ is the effect size, z_1 −α /2_ and z_1−β_ are representative for the type I and type II statistical errors and S is the pooled standard deviation. For the studies with more than two groups, the calculated N should be multiplied by $\sqrt{k-1}$ where k is the number of understudied groups.^2^ So it seems the calculated sample size is inappropriate.

The second point is about the statistical analysis. The authors mentioned “ For assessing the differences among groups at baseline and end of the study, the Kruskal-Wallis test and Chi-Square test were used for categorical and numerical variables. Wilcoxon Signed Ranks Test was used for assessing within-group changes for non-normal distribution of data”. In many randomized clinical trials, investigators assess a quantitative variable at both baseline and at the end of the study. A change score (the post-intervention score subtracted from the baseline score) is calculated for each of the studied groups. Extreme scores at baseline generally, approach to mean value during follow up (regression to the mean).^3^ Therefore, this analysis may not be the appropriate method. On the other hand it is possible that despite the randomization, the distribution of some important confounding variables may still differ between the study groups, at the baseline (randomization insufficiency). One of the best methods to overcome this problem is analysis of covariance (ANCOVA). ANCOVA compare post intervention scores adjusted for baseline values.^4^ An additional advantage of the ANCOVA is greater statistical power (detect a treatment effect with less sample size), compared to other methods.^5^ In the current study despite the non-significant difference in baseline data (maybe due to insufficient sample size), It was better to use ANCOVA.

## Ethical Approval

 Not applicable.

## Competing Interests

 The authors declare no conflict of interest in this study.
